# Derivative of the expected supremum of fractional Brownian motion at $H=1$

**DOI:** 10.1007/s11134-022-09859-3

**Published:** 2022-08-30

**Authors:** Krzysztof Bisewski, Krzysztof Dȩbicki, Tomasz Rolski

**Affiliations:** 1 Department of Actuarial Science, University of Lausanne, UNIL-Dorigny, 1015 Lausanne, Switzerland; 2 Mathematical Institute, University of Wrocław, pl. Grunwaldzki 2/4, 50-384 Wrocław, Poland

**Keywords:** fractional Brownian motion, expected supremum, *H*-derivative, 60G17, 60G22, 60G70

## Abstract

The *H*-derivative of the expected supremum of fractional Brownian motion $\{B_H(t),t\in {\mathbb {R}}_+\}$ with drift $a\in {\mathbb {R}}$ over time interval [0, *T*]

$$\begin{aligned} \frac{\partial }{\partial H} {\mathbb {E}}\Big (\sup _{t\in [0,T]} B_H(t) - at\Big ) \end{aligned}$$

at $H=1$ is found. This formula depends on the quantity ${\mathscr {I}}$, which has a probabilistic form. The numerical value of ${\mathscr {I}}$ is unknown; however, Monte Carlo experiments suggest ${\mathscr {I}}\approx 0.95$. As a by-product we establish a weak limit theorem in *C*[0, 1] for the fractional Brownian bridge, as $H\uparrow 1$.

## Introduction

Extremes of Gaussian stochastic processes play important role in many areas of stochastic modelling, including, e.g. queueing theory, risk theory, financial mathematics. Despite a substantial research effort taken in the analysis of distributional properties of suprema of Gaussian processes, most of the available results are of asymptotic nature (as for example of the tail distribution); see e.g. [9, 14, 16, 19, 22].

In this contribution we consider the expected supremum of fractional Brownian motion with drift $a\in {\mathbb {R}}$ over time horizon $T>0$, that is

$$\begin{aligned} {\mathscr {M}}_H(T,a) := {\mathbb {E}}\Big (\sup _{t\in [0,T]} B_H(t) - at\Big ), \end{aligned}$$

where $\{B_H(t),t\in {\mathbb {R}}_+\}$, with ${\mathbb {R}}_+:=[0,\infty )$, is a fractional Brownian motion with Hurst index $H\in (0,1]$ (or *H*-fBm), that is, a centred Gaussian process with the covariance function

1 $$\begin{aligned} c_H(t,s) := \mathop {\mathrm {{\mathbb {C}}ov}}\limits (B_H(t),B_H(s)) = \frac{1}{2}\left( s^{2H}+t^{2H}-|t-s|^{2H}\right) \end{aligned}$$

for all $s,t\in {\mathbb {R}}_+$. It is noted that due to *self-similarity* and *long range dependence* property (for $H>1/2$) the class of fractional Brownian motions takes a notable place in modelling of many phenomena in applied probability, as e.g. traffic in modern telecommunication networks (e.g. [17, 21]), oceanography (e.g. [24]), geophysics (e.g. [18, 20]), finance (e.g. [23]). We also refer to [13, 14] for the overview of applications and simulation techniques for *H*-fBm.

The functional ${\mathscr {M}}_H(T,a)$ plays an important role in the theory of Gaussian-driven queueing models [10–12, 15, 19, 21, 25]. More precisely, consider a single-node queue with infinite buffering capacity. Let *c* be the service rate and $\{B_H(t)+dt,t\in {\mathbb {R}}_+\}$ be the input process, that is, the traffic that enters the buffer in time interval (*s*, *t*] equals $B_H(t)+dt-(B_H(s)+ds)$. We refer to [11, 17, 25] for the formal justification that an appropriately normalized input process that is modelled by a superposition of *N* i.i.d. sources built on alternating 0-1 processes $\eta _i(t)$, i.e.

$$\begin{aligned} W_{N,T}(t):=\int _0^{tT} \sum _{i=1}^N \eta _i(s)ds \end{aligned}$$

with regularly varying tail distributions with indices in (1, 2) of the alternating epoch-times of receiving the traffic with intensity 0 or 1, respectively, and as $N\rightarrow \infty$ and $T\rightarrow \infty$, weakly converges to fractional Brownian motion with $H\in (1/2,1)$. Interestingly, for the same model but as $N\rightarrow \infty$ but $T\rightarrow 0$, the limiting process is always a fractional Brownian motion with $H=1$, see [11]. For any $T>0$, and $a:=c-d$ the buffer content process $\{Q(t),t\in {\mathbb {R}}_+\}$ satisfies the following equation

2 $$\begin{aligned} Q(T)=\max \left( Q(0)+B_H(T)-aT,\sup _{0\le s\le T}(B_H(T)-B_H(s)-a(T-s))\right) . \end{aligned}$$

Suppose now that $Q(0)=0$. Then, by *time-reversibility* of fractional Brownian motion,

$$\begin{aligned} Q(T)\overset{\mathrm{d}}{=}\sup _{t\in [0,T]} B_H(t) - at \end{aligned}$$

and hence

$$\begin{aligned} {\mathbb {E}}\left( Q(T) \right) = {\mathscr {M}}_H(T,a). \end{aligned}$$

**Fig. 1:**
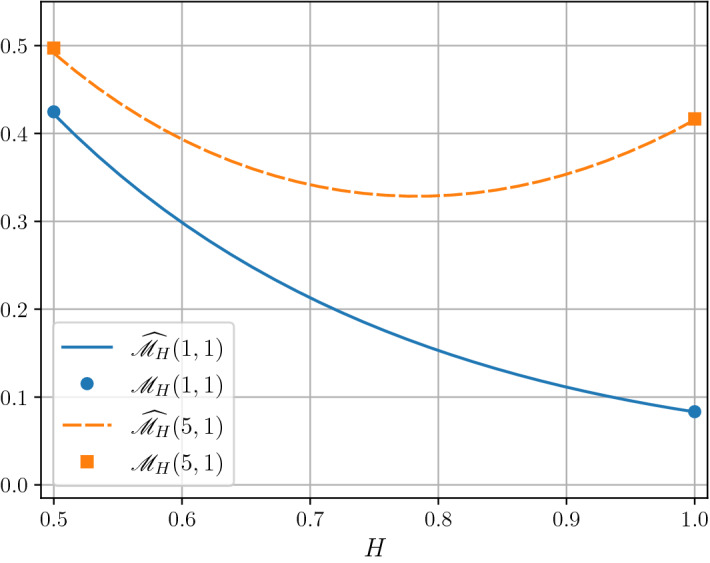
Numerical results for the estimation of ${\mathscr {M}}_H(T,1)$ for $T=1$ and $T=5$ for all $H\in [\tfrac{1}{2},1]$. The estimates are based on Monte Carlo simulations of fBm with $2\cdot 10^{5}$ independent samples on an equispaced grid with $2^{16}$ gridpoints. The half-widths of $95\%$ confidence intervals are at most 0.00086 and 0.0038 in cases $T=1$ and $T=5$ correspondingly. The markers (filled circle and a square) correspond to the theoretical values of ${\mathscr {M}}_H(T,a)$ known only in cases $H=\tfrac{1}{2}$ and $H=1$, cf.(4)

In this paper we continue our studies of the *H*-derivative of the expected supremum from [6], that is, we consider

$$\begin{aligned} {\mathscr {M}}_H'(T,a) := \frac{\partial }{\partial H} {\mathscr {M}}_H(T,a), \end{aligned}$$

focusing on the case $H=1$. More specifically, in Theorem  1, which presents the main result of this contribution, we derive the formula for ${\mathscr {M}}_1'(T,a)$. One of motivations for our studies, which arose from the analysis of simulations of ${\mathscr {M}}_H(T,a)$, is its behaviour for *H* close to 1. There is some indication that for sufficiently large *T*, ${\mathscr {M}}_H(T,a)$ as function of *H* has a U-shape in some sub-interval (0, *b*), where $b<1$ This observation is supported by the fact that by self-similarity of fBm ${\mathscr {M}}_H(T,0) = T^H {\mathscr {M}}_H(1,0)$, so for *T* large enough we should observe this phenomenon. On Fig. 1 we show simulated functions $H\mapsto {\mathscr {M}}_H(T,a)$ for $a=1$ and $T=1\ {\text{ and }\ T=5}$.

The findings of this contribution answer some of these questions. In particular, Theorem 1 implies that there is a threshold value of the model parameter *T* which distinguishes between scenarios when ${\mathscr {M}}_1'(T,a)$ is negative, equal to 0 or positive, and which (unexpectedly) does not depend on the drift parameter *a*. Hence, it follows that in the neighbourhood of $H=1$ the function $H\mapsto {\mathscr {M}}_H(T,a)$ can be decreasing or increasing depending on whether *T* is small or big. Moreover, knowing the derivative, we may approximate $M_H(T,a)$ for *H* close to 1, i.e.

$$\begin{aligned} {\mathscr {M}}_H(T,a) = {\mathscr {M}}_1(T,a) - (1-H){\mathscr {M}}'_1(T,a) + o(1-H) \end{aligned}$$

This gives one of motivations to study the derivative at $H=1$.

This paper complements our previous work [6], where we focused on the case $H=\tfrac{1}{2}$ and found that

3 $$\begin{aligned} {\mathscr {M}}'_{1/2}(T,a) = \frac{1}{\sqrt{\pi }|a|} \left( \log (2a^{-2})\gamma (\tfrac{1}{2},\tfrac{a^2T}{2}) +\gamma '(\tfrac{1}{2},\tfrac{a^2T}{2})\right) , \end{aligned}$$

where $\gamma '(s,x) := \frac{\partial }{\partial s}\gamma (s,x)$ and $\gamma (s,x) := \int _0^x t^{s-1}e^{-t}\mathrm{d}t$ is the lower incomplete gamma function; see [6, Theorem 1] and also [4, Corollary 4]. The values of ${\mathscr {M}}'_{1/2}(T,a)$ in the cases $a=0$ and $T=\infty$ can be found by passing to the limit in the formula above with $a\rightarrow 0$ and $T\rightarrow \infty$, respectively. See also [6, Corollary 1(i-ii)].

We now make a small survey of what is known about ${\mathscr {M}}_H(T,a)$. Even though the quantity ${\mathscr {M}}_H(T,a)$ is so fundamental, its value is known explicitly only in two special cases $H=\tfrac{1}{2}$ and $H=1$,

4 $$\begin{aligned} {\mathscr {M}}_{1/2}(T,a)= & {} \frac{1}{2a}\left( -a^2T + (1+ a^2T)\mathop {\mathrm {erf}}\limits \Big (a\sqrt{\tfrac{T}{2}}\Big )+ \sqrt{\frac{2T}{\pi }}\cdot ae^{-a^2T/2}\right) \nonumber \\ {\mathscr {M}}_1(T,a)= & {} T \cdot \left( \frac{1}{\sqrt{2\pi }}e^{-a^2/2} - \frac{a}{2}\mathop {\mathrm {erfc}}\limits \Big (\frac{a}{\sqrt{2}}\Big )\right) , \end{aligned}$$

where $\mathop {\mathrm {erf}}\limits (\cdot )$ and $\mathop {\mathrm {erfc}}\limits (\cdot )$ is the error and complementary error functions, respectively. The derivation of ${\mathscr {M}}_{1/2}(T,a)$ can be found, e.g. in [4, Proposition 2] while the formula for ${\mathscr {M}}_1(T,a)$ can be calculated straightforwardly using the fact that ${\mathscr {M}}_1(T,a) = {\mathbb {E}}(B_1(T)-aT)_+$, where $z_+ := \max \{z,0\}$. The borderline cases $a=0$ and $T=\infty$ can be found by passing to the limit in the formulas above as $a\rightarrow 0$ or $T\rightarrow \infty$, respectively.

Recently, properties of function $H\mapsto {\mathscr {M}}_H(T,a)$ attracted notable attention. First, from Sudakov–Fernique’s inequality it straightforwardly follows that $H\mapsto {\mathscr {M}}_H(1,0)$ is an non-increasing function. However, note that it need not to be true for $T>1$. Second, due to Borovkov *et al* [7, 8], with a recent improvement by Bisewski [4],

$$\begin{aligned} 1.128\le H^{1/2} {\mathscr {M}}_H(1,0)\le 1.695 \end{aligned}$$

for sufficiently small *H*, which supports the conjecture that there exists a constant $C\in (0,\infty )$ such that

$$\begin{aligned} C=\lim _{H\downarrow 0}H^{1/2} {\mathscr {M}}_H(1,0). \end{aligned}$$

We also refer to [5] for the analysis of ${\mathscr {M}}_H(\infty ,a)$ as a function of *H*, with $a>0$.

*Organization of the paper:* in Sect. 2 we derive some useful properties of fractional Brownian bridges, that will play important role in the proof of the main result, which is given in Sect. 3. In Proposition 1 we establish a weak limit theorem in *C*[0, 1] for the fractional Brownian bridge, as $H\uparrow 1$. The main result is given in Theorem 1. All the proofs are postponed to Sect. 4.

## Fractional Brownian bridge and its limit

In this section, we derive some properties of fractional Brownian bridges, that will play crucial role in the proofs of the main result of this contribution. The main result is the limit in distribution at $H=1$.

Let $\{B^0_H(t) : t\in [0,1]\}$ be a fractional Brownian bridge (fBB), that is, an fBm pinned at $B_H(1) = 0$, which is defined by conditioning

$$\begin{aligned} \{B_H^0(t), t\in [0,1]\} \overset{\mathrm{d}}{=}\left\{ B_H(t) \mid B_H(1)=0, t\in [0,1]\right\} . \end{aligned}$$

It is noted that applying the standard formula for the distribution of the multivariate Gaussian vector conditioned by the value of a given coordinate (see, e.g. Introduction in [22]), we have

$$\begin{aligned} \mathop {\mathrm {{\mathbb {C}}ov}}\limits (B^0_H(t),B^0_H(s)) = c_H(t,s)-c_H(t,1)c_H(s,1) \end{aligned}$$

and the following equality in distribution holds

$$\begin{aligned} B^0_H(t) \overset{\mathrm{d}}{=}B_H(t)-c_H(t,1)B_H(1), \qquad 0\le t\le 1. \end{aligned}$$

Analogously, the fBm pinned at $B_H(1) = x$, which is defined by conditioning

$$\begin{aligned} \{B_H^{{x}}(t), t\in [0,1]\} \overset{\mathrm{d}}{=}\left\{ B_H(t) \mid B_H(1)=x, t\in [0,1]\right\} . \end{aligned}$$

follows the representation

5 $$\begin{aligned} B_H^x(t) \overset{\mathrm{d}}{=}B^0_H(t)+xc_H(t,1), {\qquad 0\le t\le 1}. \end{aligned}$$

When $H=1$, the fBB becomes a deterministic straight line from (0, 0) to (1, 0). However, it turns out that if we blow up this process by factor $(1-H)^{-1/2}$, its distribution converges to a non-trivial limit as $H\uparrow 1$. More precisely, for every $H\in (0,1)$ let

6 $$\begin{aligned} X_H(t) := \frac{B_H^0(t)}{\sqrt{1-H}}, \quad t\in [0,1]. \end{aligned}$$

In the following let $\{X(t),t\in [0,1]\}$ be a centred Gaussian bridge with $X(0)=X(1)=0$ and the covariance function

7 $$\begin{aligned}&\mathop {\mathrm {{\mathbb {C}}ov}}\limits (X(t),X(s)) := g(t,s) - tg(1,s) - sg(1,t) \end{aligned}$$

8 $$\begin{aligned}&g(t,s) := -\left( t^2\log (t) + s^2\log (s) - |t-s|^2\log |t-s|\right) , \end{aligned}$$

where we follow the convention that $0^2\log (0) := \lim _{t\rightarrow 0^+}t^2\log (t) = 0$. We remark that

$$\begin{aligned} \mathop {\mathrm {{\mathbb {V}}ar}}\limits (X(t)) = g(t,t)-2g(t,1) = -2t(1-t)\left( t\log (t) + {(1-t)}\log (1-t)\right) . \end{aligned}$$

It is also noted that (7) constitutes a covariance function, since the limit of positively definite functions is a positively definite function.

### Proposition 1

The scaled fractional Brownian bridge $\left\{ X_H(t): t\in [0,1]\right\}$ converges weakly in space *C*[0, 1] to $\{X(t) : t\in [0,1]\}$, as $H\uparrow 1$.

We postpone the proof of Proposition 1 to Sect. 4.

## The main theorem

Before proceeding to the main result of this paper, we note that by simple time-reversal argument we can find that $M_H(T,-a) \overset{\mathrm{d}}{=}M_H(T,a) - (B_H(T) - aT)$. Thus for any $T\in {\mathbb {R}}_+$, $a\in {\mathbb {R}}$ we have

$$\begin{aligned} {\mathscr {M}}_H(T,-a) = {\mathscr {M}}_H(T,a) + Ta \end{aligned}$$

and therefore, provided that ${\mathscr {M}}'_H(T,a)$ exists, we have

$$\begin{aligned} {\mathscr {M}}'_H(T,-a) = {\mathscr {M}}'_H(T,a). \end{aligned}$$

Let

9 $$\begin{aligned} {\mathscr {I}} := {\mathbb {E}}\left( \int _{-\infty }^\infty \sup _{t\in [0,1]} \left\{ X(t)+tz - z_+\right\} \mathrm{d}z\right) \end{aligned}$$

In the following, the standard normal probability density function is denoted by $\phi (\cdot )$.

### Theorem 1

For any $T>0$ and $a\in {\mathbb {R}}$ it holds that

$$\begin{aligned} {\mathscr {M}}_1'(T,a) = T(\log (T)-{\mathscr {I}})\phi (a) \end{aligned}$$

and $${\mathscr {I}} \in (0,\infty ).$$

The proof of Theorem 1 is postponed to Sect. 4.

### Remark 1

It is noted that ${\mathscr {M}}_1(\infty ,a) = \infty$ for all $a\in {\mathbb {R}}$; see e.g. [5] and therefore ${\mathscr {M}}'_1(\infty ,a)$ does not exist. Hence, intuitively it is clear that one should expect ${\mathscr {M}}_1'(T,a)>0$ for sufficiently large *T*. Indeed, it straightforwardly follows from Theorem 1 that the criterion for the sign of ${\mathscr {M}}_1'(T,a)$ is *T* to be smaller or larger than $\exp ({\mathscr {I}})$.

### Remark 2

Using the fact that the process *X*(*t*) is time-reversible, it is easy to see that

$$\begin{aligned} {\mathscr {I}} = 2\,{\mathbb {E}}\left( \int _0^\infty \sup _{t\in [0,1]} \left\{ X(t)-tz\right\} \mathrm{d}z\right) . \end{aligned}$$

One can recognize that the function $z\mapsto \sup _{t\in [0,1]}\{X(t) - tz\}$ is the *convex conjugate* (or *Legendre–Fenchel transformation*) of a random trajectory $t\mapsto X(t)$. While we were not able to calculate the theoretical value of ${\mathscr {I}}$, our numerical experiments strongly suggest that ${\mathscr {I}} \approx 0.95$.

**Fig. 2:**
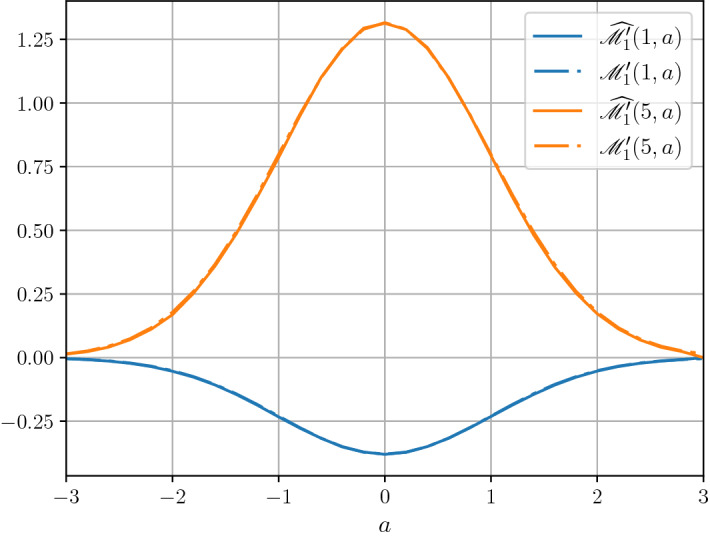
Numerical results for the estimation of ${\mathscr {M}}'_1(T,a)$ for $T=1$ and $T=5$ for all $a\in [-3,3]$. The half-widths of $95\%$ confidence intervals are at most 0.0020 and 0.013 in cases $T=1$ and $T=5$ correspondingly. We observe that, on this scale, it is hard to distinguish between the numerical and the theoretical results

In Fig. 2 we present numerical results for the estimation of ${\mathscr {M}}'_1(T,a)$ for $T=1$ and $T=5$ for all $a\in [-3,3]$. The estimates are based on Monte Carlo simulations of fBm with $10^{6}$ independent samples on an equispaced grid with $2^{16}$ gridpoints. The dashed lines are the estimates while the solid lines correspond to the theoretical values derived in Theorem 1 using the approximation that ${\mathscr {I}} = 0.95$. Note that the time horizons *T* were chosen such that ${\mathscr {M}}_1'(1,a) < 0$ and ${\mathscr {M}}_1'(5,a) > 0$ for all *a*.

*Concluding remarks.*

In this contribution we have analysed the first derivative of $M_H(T,a)$ at $H=1$. We suspect that the use of similar techniques can give some insight into derivatives of higher moments of $\sup _{t\in [0,T]} B_H(t) - at$ at $H=1$, which however looks to be more technically challenging. There are also some hopes that the line of argumentation used in this contribution can be useful for the analysis of higher derivatives of $M_H(T,a)$ at $H=1$. Complementary results, for $H=1/2$ have been obtained in [6], with the use of tools that work for processes close to Brownian motion (that is around $H=1/2$) and hence are different than applied in this paper.

## Proofs

In this section we provide detailed proofs of the findings presented in this contribution. We begin with lemma that is useful in the proof of Proposition 1.

### Lemma 1

There exists ${\mathcal {C}}>0$ such that

$$\begin{aligned} {\mathbb {E}}\left( X_H(t) - X_H(s)\right) ^2 \le {\mathcal {C}}|t-s|^{2H}|\log |t-s||, \quad t,s\in [0,1] \end{aligned}$$

for all $$H\in (\tfrac{1}{2},1)$$.

### Proof of Lemma 1

Till the end of the proof, without loss of generality, we assume that $s<t$. Utilizing that, for any $t,s\in [0,1]$,

10 $$\begin{aligned} {\mathbb {E}}\left( B^0_H(t) - B^0_H(s)\right) ^2 = |t-s|^{2H} - \tfrac{1}{4}f^2_H(t,s), \end{aligned}$$

where

$$\begin{aligned} f_H(t,s) := t^{2H}-(1-t)^{2H} + (1-s)^{2H} - s^{2H}, \end{aligned}$$

by subtracting and adding $(t-s)^{2H}$ to (10) we obtain the following bound

11 $$\begin{aligned} {\mathbb {E}}\left( \frac{B^0_H(t)}{\sqrt{1-H}} - \frac{B^0_H(s)}{\sqrt{1-H}}\right) ^2 \le \frac{|(t-s)^{2H}-(t-s)^2|}{1-H} + \frac{|\tfrac{1}{4}f_H^2{(t,s)}-(t-s)^2|}{1-H}. \end{aligned}$$

After applying the mean value theorem (with respect to *H*) to the first term, we obtain

12 $$\begin{aligned} \frac{|(t-s)^{2H}-(t-s)^2|}{1-H} \le \sup _{{\widetilde{H}}\in [H,1]}2(t-s)^{2{\widetilde{H}}}|\log (t-s)| = 2(t-s)^{2H}|\log (t-s)|. \end{aligned}$$

Similarly, noting that $f_1(t,s) = 2(t-s)$ we apply the mean value theorem to the second term in (11) which, with $f'_H(t,s) := \frac{\partial }{\partial H} f_H(t,s)$, yields

13 $$\begin{aligned} \frac{|\tfrac{1}{4}f^2_H(t,s)-(t-s)^2|}{1-H} \le \tfrac{1}{2}\sup _{{\widetilde{H}}\in [H,1]} |f_{{\widetilde{H}}}(t,s) \cdot f'_{{\widetilde{H}}}(t,s)|. \end{aligned}$$

Since

$$\begin{aligned} f_H(t,s) = w_H(t)-w_H(s), \qquad w_H(\theta ):=\theta ^{2H} - (1-\theta )^{2H} \end{aligned}$$

and similarly

$$\begin{aligned} f'_H(t,s) = w'_H(t)-w'_H(s), \qquad w'_H(\theta )=2\left( \theta ^{2H}\log (\theta ) - (1-\theta )^{2H}\log (1-\theta )\right) . \end{aligned}$$

we may apply the mean value theorem again (but now with respect to *s*), which yields

14 $$\begin{aligned} \frac{|f_H(t,s)|}{|t-s|} \le \sup _{\theta \in [s,t]} \left| \frac{\partial }{\partial \theta } w_H(\theta )\right| , \qquad \frac{|f'_H(t,s)|}{|t-s|} \le \sup _{\theta \in [s,t]} \left| \frac{\partial }{\partial \theta } w'_H(\theta )\right| . \end{aligned}$$

Finally, we have

$$\begin{aligned} \frac{\partial }{\partial \theta } w_H(\theta ) = 2H\theta ^{2H-1} + 2H(1-\theta )^{2H-1}, \end{aligned}$$

and therefore $\frac{\partial }{\partial \theta } w_H(\theta ) \le 4$ for all $H\in [1/2,1)$. Similarly,

$$\begin{aligned} \frac{\partial }{\partial \theta } w'_H(\theta ) = z_H(\theta ) + z_H(1-\theta ), \qquad z_H(x) := 2\left( x^{2H-1} + 2Hx^{2H-1}\log (x)\right) , \end{aligned}$$

and therefore

$$\begin{aligned} \sup _{\theta \in [s,t]} |w_H'(\theta )| \le 2 \sup _{x\in [0,1]} |z_H(x)| \le 4 +4\sup _{x\in [0,1]} x^{2H-1}|\log (x)|. \end{aligned}$$

Now, it is clear that there exists some $C>0$ such that $\sup _{x\in [0,1]} x^{2H-1}|\log (x)| \le C$ for all $H<1$ large enough. Therefore, using (14) and going back to (13) we obtain

$$\begin{aligned} \frac{|\tfrac{1}{4}f^2_H(t,s)-(t-s)^2|}{1-H} \le \tfrac{1}{2} \big (4(t-s)\big ) \big (4(1+C)(t-s)\big ) = 8(1+C)|t-s|^2. \end{aligned}$$

Finally, the bound above combined with (12) and (11) yields

$$\begin{aligned} {\mathbb {E}}\left( \frac{B^0_H(t)}{\sqrt{1-H}} - \frac{B^0_H(s)}{\sqrt{1-H}}\right) ^2 \le 2(t-s)^{2H}|\log (t-s)| + 8(1+C)|t-s|^2, \end{aligned}$$

which concludes the proof. $\square$

### Proof of Proposition 1

First we will show that finite dimensional distributions (fdds) of $X_H$ converge to those of *X*, which in case of centred Gaussian processes is equivalent to the convergence of the covariance function, i.e.

15 $$\begin{aligned} \mathop {\mathrm {{\mathbb {C}}ov}}\limits (X_H(t),X_H(s))\rightarrow {\mathop {\mathrm {{\mathbb {C}}ov}}\limits (X(t),X(s)),} \quad H\uparrow 1 \end{aligned}$$

for all $t,s\in [0,1]$. Moreover, due to Lemma 1, the conditions of Theorem 12.3 from Billingsley [2] are satisfied (see also Eq. (12.51) immediately below Theorem 12.3) and therefore the sequence $X_H$ is tight in *C*[0, 1]. This, together with the convergence of fdds demonstrated below would complete the proof of the weak convergence.

Till this end we will show (15). Using Taylor expansion (at $H=1$), for every fixed $t\in (0,1)$ we have

$$\begin{aligned} t^{2H} = t^2 - 2(1-H)t^2\log (t) + o(1-H), \end{aligned}$$

as $H\uparrow 1$. Without loss of generality assume that $0<s<t<1$. The proof in case $s=t$ is analogous and slightly simpler. Using the notation for function *g*(*t*, *s*) introduced in (8) we have

$$\begin{aligned} c_H(t,s) = ts + (1-H)g(t,s) + o(1-H). \end{aligned}$$

Thus

$$\begin{aligned}&\mathop {\mathrm {{\mathbb {C}}ov}}\limits (X_H(t),X_H(s)) = \frac{c_H(t,s) - c_H(t,1)c_H(s,1)}{1-H} \\&= \frac{ts + (1-H)g(t,s) - \big (t + (1-H)g(1,t)\big )\big (s + (1-H)g(1,s)\big )}{1-H} + o(1), \end{aligned}$$

which implies that $\mathop {\mathrm {{\mathbb {C}}ov}}\limits (X_H(t),X_H(s)) = g(t,s) - tg(1,s)-sg(1,t) + o(1)$ and concludes the proof. $\square$

### Lemma 2

For every $\varepsilon \in (0,1)$ there exists a random variable $\kappa _{H,\varepsilon }$, which satisfies

$$\begin{aligned} |X_H(t)-X_H(s)| \le \kappa _{H,\varepsilon }|t-s|^{1-\varepsilon }, \quad t,s\in [0,1] \end{aligned}$$

for all $H\in (1-\tfrac{\varepsilon }{2},1)$. Moreover, for every $\varepsilon ,p>0$ there exists a finite constant ${\mathcal {K}}:={\mathcal {K}}(\varepsilon ,p)$ such that

$$\begin{aligned} \sup _{H\in (1-\varepsilon /2,1)} {\mathbb {E}}|\kappa _{H,\varepsilon }|^p \le {\mathcal {K}}. \end{aligned}$$

### Proof

Using Lemma 1 we find that for $t,s\in [0,1]$ we have

$$\begin{aligned} \sqrt{{\mathbb {E}}(X_H(t)-X_H(s))^2}&\le \sqrt{{\mathcal {C}}} |t-s|^{1-\varepsilon } \cdot |t-s|^{H-1+\varepsilon }\sqrt{|\log |t-s||} \\&\le \sqrt{{\mathcal {C}}} |t-s|^{1-\varepsilon } \cdot \sqrt{|t-s|^\varepsilon |\log |t-s||}, \end{aligned}$$

where in the last line we used $H>1-\tfrac{\varepsilon }{2}$. It can be seen that for every $\sup _{t\in [0,1]} t^\varepsilon |\log (t)| < \infty$ for every $\varepsilon >0$, therefore for every $\varepsilon >0$ there exists ${\mathcal {C}}_\varepsilon$ such that

$$\begin{aligned} \sqrt{{\mathbb {E}}(X_H(t)-X_H(s))^2} \le {\mathcal {C}}_\varepsilon |t-s|^{1-\varepsilon }, \quad t,s\in [0,1]. \end{aligned}$$

for all $H\in (1-\tfrac{\varepsilon }{2},1)$. The first part of Lemma 2 now follows from [1, Theorem 1]. The fact that ${\mathcal {K}}$ can be chosen uniformly for al $H\in (1-\tfrac{\varepsilon }{2},1)$ is implicit from the proof of [1, Theorem 1]. In particular, it follows from the fact that the constant ${\mathcal {C}}_\varepsilon$ above is chosen uniformly for all $H\in (1-\tfrac{\varepsilon }{2},1)$ and that the constant in the Garsia–Rodemich–Rumsey inequality used in the proof of [1, Theorem 1] depends only on $\varepsilon$. $\square$

Before we show the proof of Theorem 1, we need one technical result. In the following, let

16 $$\begin{aligned} \ell _H(t,z,a) := c_H(t,1)z-z_+ + \frac{c_H(t,1)-t}{\sqrt{1-H}} \cdot a. \end{aligned}$$

### Lemma 3

There exists $L>0$ such that for all $t\in [0,1]$, $z,a\in {\mathbb {R}}$

(i) $$\ell _H(t,z,a) \le L|a|\sqrt{1-H}$$;
(ii) $$|\ell _H(t,z,a)-(tz - z_+)| \le L(1+|z|)\sqrt{1-H}\cdot t(1-t)$$

for all $H<1$ sufficiently large.

### Proof of Lemma 3

With no loss of generality, suppose that $H>3/4$. We begin with the proof that there exists $L>0$ such that for all $H\in [3/4,1]$ and $t\in [0,1]$

17 $$\begin{aligned} | c_H(t,1)-t| \le L(1-H)t(1-t). \end{aligned}$$

Following Taylor expansion of function $k_t(H):=c_H(t,1)-t$ with respect to *H* at point $H=1$, we have that for each $t\in [0,1]$ there exists $H_t\in [H,1]\subset [3/4,1]$ such that

$$\begin{aligned} |k_t(H)|= & {} |(1-H)k'_t(H_t)| = (1-H)\left| \log (t)t^{2H_t}-\log (1-t)(1-t)^{2H_t}\right| \\\le & {} (1-H) \left( |\log (t)|t^{2H_t}+|\log (1-t)|(1-t)^{2H_t}\right) \\\le & {} (1-H) \left( |\log (t)|t^{3/2}+|\log (1-t)|(1-t)^{3/2}\right) . \end{aligned}$$

Next, using that $\sup _{t\in (0,1]}|\log (t)|t^{1/2}<\infty$, $\lim _{t\rightarrow 0} |\log (t)|t^{1/2}=0$ and $|\log (t)|t^{1/2}=(1-t)(1+o(1-t))$ as $t\uparrow 1$, we conclude that there exists a constant $L>0$ such that $|\log (t)|t^{1/2}\le \frac{L}{2} (1-t)$ for all $t\in (0,1]$, which implies that

$$\begin{aligned} |\log (t)|t^{3/2}\le \frac{L}{2}(1-t)t \end{aligned}$$

for all $t\in (0,1]$. Similarly, for all $t\in [0,1)$, we get

$$\begin{aligned} |\log (1-t)|(1-t)^{3/2}\le \frac{L}{2} (1-t)t. \end{aligned}$$

This leads to the conclusion that

$$\begin{aligned} |k_t(H)|\le L(1-H)t(1-t) \end{aligned}$$

and hence (17) holds.

Ad. (i). Using that $c_H(t,1)z-z_+\le 0$ for all $t\in [0,1]$, $z\in {\mathbb {R}}$ and $t(1-t)\le 1/4$ for $t\in [0,1]$, (i) follows straightforwardly from (17).

Ad. (ii). Observe that

$$\begin{aligned} |\ell _H(t,z,a)-(tz - z_+)| \le |z| | c_H(t,1)-t| +\frac{|a|}{\sqrt{1-H}} |c_H(t,1)-t|. \end{aligned}$$

Now application of (17) implies (ii). $\square$

### Proof of Theorem 1

Recall that $z_+ := \max \{z,0\}$. Using self-similarity and the fact that $\{B_1(t):t\in [0,T]\} \overset{\mathrm{d}}{=}\{t\cdot T^{-H}B_H(T):t\in [0,T]\}$ and $\sup _{t\in [0,T]} \{B_1(t) - at\} \overset{d}{=} (T^{1-H}B_H(T)-aT)_+$, we have

18 $$\begin{aligned} \frac{{\mathscr {M}}_H(T,a)-{\mathscr {M}}_1(T,a)}{H-1}&= {\mathbb {E}}\left( \frac{\sup _{t\in [0,T]} \{B_H(t)-at\} - (T^{1-H}B_H(T)-aT)_+\}}{H-1}\right) \nonumber \\&= {\mathbb {E}}\left( \frac{\sup _{t\in [0,1]} \{T^HB_H(t)-aTt\} - (TB_H(1)-aT)_+}{H-1}\right) \nonumber \\&= {\mathcal {D}}^{(1)}_H(T,a) + {\mathcal {D}}^{(2)}_H(T,a), \end{aligned}$$

with

$$\begin{aligned} {\mathcal {D}}^{(1)}_H(T,a)&:= -T^{H}{\mathbb {E}}\left( \frac{\sup _{t\in [0,1]} \{B_H(t)-aT^{1-H}t\} - (B_H(1)-aT^{1-H})_+}{1-H}\right) \\ {\mathcal {D}}^{(2)}_H(T,a)&:= T \cdot \frac{{\mathbb {E}}(B_H(1)-a)_+ - {\mathbb {E}}(T^{H-1}B_H(1)-a)_+}{1-H}. \end{aligned}$$

In (18) we simply added and subtracted $(T^HB_H(1)-aT)_+$ in the numerator. First, it can be straightforwardly calculated that

$$\begin{aligned} \lim _{H\rightarrow 1}{\mathcal {D}}^{(2)}_H(T,a) = T\log (T)\phi (a). \end{aligned}$$

We now focus on ${\mathcal {D}}^{(1)}_H(T,a)$. We have

$$\begin{aligned}&{\mathcal {D}}^{(1)}_H(T,a)\\&= -T^H\int _{-\infty }^\infty {\mathbb {E}}\left( \sup _{t\in [0,1]} \frac{B_H(t)-aT^{1-H}t- T^{1-H}(x-a)_+}{1-H}\mid B_H(1)=x\right) \phi (x)\mathrm{d}x \\&= -T^{2H-1}\int _{-\infty }^\infty {\mathbb {E}}\left( \sup _{t\in [0,1]} \frac{B_H(t)-aT^{1-H}t - y_+}{1-H}\mid B_H(1) = a + T^{H-1}y\right) \phi (a + T^{H-1}y)\mathrm{d}y, \end{aligned}$$

where we substituted $x = a + T^{H-1}y$. Using (5) we can write

$$\begin{aligned}&= -T^{2H-1}\int _{-\infty }^\infty {\mathbb {E}}\left( \sup _{t\in [0,1]} \frac{B^0_H(t)+c_H(t,1)(y+aT^{1-H}) - y_+}{1-H}\right) \phi (y+aT^{H-1})\mathrm{d}y \end{aligned}$$

and after applying the substitution $y = \sqrt{1-H}z$, we obtain

19 $$\begin{aligned} \frac{{\mathscr {M}}_H(T,a)-{\mathscr {M}}_1(T,a)}{H-1} = -T^{2H-1}\phi (a) \int _{-\infty }^\infty f_H(z)r_H(z)\mathrm{d}z, \end{aligned}$$

where

$$\begin{aligned} f_H(z):={\mathbb {E}}\sup _{t\in [0,1]} \left\{ X_H(t)+\ell _H(t,z,a)\right\} \cdot r_H(z), \quad r_H(z,a):=\frac{\phi (\sqrt{1-H}z+aT^{H-1})}{\phi (a)} \end{aligned}$$

with process $X_H(t)$ defined in (6) and the function $\ell _H(t,z,{a})$ defined in (16).

Till this end we will show that $\int _{-\infty }^\infty f_H(z)r_H(z)\mathrm{d}z \rightarrow {\mathscr {I}}$, as $H\uparrow 1$. Lemma 3(ii) implies that for every fixed $z\in {\mathbb {R}}$, the function $t\mapsto \ell _H(t,z,a)$ converges uniformly to $t\mapsto tz - z_+$ on [0, 1], as $H\uparrow 1$. This observation combined with the result in Proposition 1 implies that $\left\{ X_H(t) + \ell _H(t,z,a): t\in [0,1]\right\}$ converges weakly to $\{X(t) +tz - z_+: t\in [0,1]\}$ in *C*[0, 1] space, as $H\uparrow 1$. By the virtue of continuity of $\sup$ functional in *C*[0, 1] space, we conclude that

20 $$\begin{aligned} \sup _{t\in [0,1]} \left\{ X_H(t)+\ell _H(t,z,a)\right\} \overset{\mathrm{d}}{\rightarrow }\sup _{t\in [0,1]} \left\{ X(t)+tz-z_+\right\} , \quad H\uparrow 1. \end{aligned}$$

Till this end, let $\varepsilon \in (0,\tfrac{1}{2})$. Combining Lemma 3(i) with Lemma 2 we find that for any $p\ge 1$ and $z\in {\mathbb {R}}$ it holds that

21 $$\begin{aligned} {\mathbb {E}}\Big |\sup _{t\in [0,1]} \left\{ X_H(t)+\ell _H(t,z,a)\right\} \Big |^p&\le {\mathbb {E}}\Big |(\kappa _{H,\varepsilon }) + L|a|\sqrt{1-H}\Big |^p \nonumber \\&\le 2^{p-1}\left( {\mathbb {E}}|\kappa _{H,\varepsilon }|^p + (L|a|\sqrt{1-H})^p\right) , \end{aligned}$$

for all $H<1$ sufficiently large. In the second line we used the inequality $(a+b)^p \le 2^{p-1}(a^p + b^p)$, which holds for any $a,b>0$, $p\ge 1$. Moreover by the virtue of the second part of Lemma 2, there exists a finite $\kappa$ such that ${\mathbb {E}}|\kappa _{H,\varepsilon }|^{1/\varepsilon } < {\mathcal {K}}$ for all $H<1$ large enough. To conclude this part of the proof, for every $p>1$ there exists some finite $C'_p>0$ such that

22 $$\begin{aligned} {\mathbb {E}}\Big |\sup _{t\in [0,1]} \left\{ X_H(t)+\ell _H(t,z,a)\right\} \Big |^p < C'_p, \quad z\in {\mathbb {R}}\end{aligned}$$

for all $H<1$ large enough. Using Lemma 3(ii) we obtain

$$\begin{aligned} \sup _{t\in [0,1]} \left\{ X_H(t)+\ell _H(t,z,a)\right\} \le \sup _{t\in [0,1]} \left\{ X_H(t) + zt - z_+ + L(1+|z|)\sqrt{1-H}t(1-t)\right\} . \end{aligned}$$

Assume for a moment that $z\ge 0$, then $zt - z_+ = -(1-t)|z|$ and using the fact that the process $X_H(t)$ is reversible in [0, 1], i.e. $\{X_H(t):t\in [0,1]\} \overset{\mathrm{d}}{=}\{X_H(1-t):t\in [0,1]\}$ (which can be easily proven by direct calculation of the covariance function of fBB); therefore, the upper bound above is equal in distribution to

$$\begin{aligned} \sup _{t\in [0,1]} \left\{ X_H(t) -|z|t + c(1+|z|)\sqrt{1-H}t(1-t)\right\} . \end{aligned}$$

Analogous upper bound holds true when $z<0$. Now, for all $H<1$ large enough we have

$$\begin{aligned} -|z|t + c(1+|z|)\sqrt{1-H}t(1-t) \le -\tfrac{1}{2}(|z|-1)t \end{aligned}$$

and using Lemma 2 we further obtain

$$\begin{aligned} \sup _{t\in [0,1]} \left\{ X_H(t)+\ell _H(t,z,a)\right\} \le \sup _{t\in [0,1]} \left\{ \kappa _{H,\varepsilon } t^{1-\varepsilon } -\tfrac{1}{2}(|z|-1)t\right\} . \end{aligned}$$

The supremum on the right-hand side above can be found explicitly, which finally yields the following upper bound (in distribution)

$$\begin{aligned} \sup _{t\in [0,1]} \left\{ X_H(t)+\ell _H(t,z,a)\right\} \le C_1 \cdot (\kappa _{H,\varepsilon })^{1/\varepsilon } (|z|-1)^{-(1-\varepsilon )/\varepsilon }, \quad |z|>1, \end{aligned}$$

where $C_1:=\varepsilon (2(1-\varepsilon ))^{(1-\varepsilon )/\varepsilon }$. Using the same reasoning as in the proof of the upper bound in (22), we conclude for any $p\ge 1$ there exists some finite $C_p''$ such that

23 $$\begin{aligned} {\mathbb {E}}\Big |\sup _{t\in [0,1]} \left\{ X_H(t)+\ell _H(t,z,a)\right\} \Big |^p \le C_p'' \cdot (|z|-1)^{-(1-\varepsilon )/\varepsilon }, \quad |z|>1 \end{aligned}$$

for all $H<1$ large enough.

Now, the bounds in (22) and (23), as functions of the variable *z* are integrable over $[-2,2]$ and ${\mathbb {R}}\setminus [-2,2]$, respectively, for all $p\ge 1$. Therefore, by combining them together we obtain a dominating, integrable function. Using the convergence in (20) and an inequality on integrable majorant, by the Corollary from page 348 of [3], we have

24 $$\begin{aligned} \lim _{H\uparrow 1} {\mathbb {E}}\sup _{t\in [0,1]}\left\{ X_H(t)+\ell _H(t,z,a)\right\} = {\mathbb {E}}\sup _{t\in [0,1]} \left\{ X(t)+tz-z_+\right\} . \end{aligned}$$

Finally, since $0 \le r_H(z) \le (\phi (a)\sqrt{2\pi })^{-1}$, $z\in {\mathbb {R}}$ we may apply the Lebesgue dominated convergence theorem to conclude that

$$\begin{aligned} \lim _{H\uparrow 1}\int _{-\infty }^\infty {\mathbb {E}}\sup _{t\in [0,1]} \left\{ X_H(t)+\ell _H(t,z,a)\right\} \cdot {r_H}(z)\mathrm{d}z = {\mathscr {I}}. \end{aligned}$$

It is left to show that ${\mathscr {I}} > 0$. By taking only

$$\begin{aligned} {\mathscr {I}}&= \int _{-\infty }^\infty {\mathbb {E}}\left( \sup _{t\in [0,1]} \left\{ X(t)+tz-z_+\right\} \right) \mathrm{d}z \\&= \int _{-\infty }^0 {\mathbb {E}}\left( \sup _{t\in [0,1]} \left\{ X(t)-|z|t\right\} \right) \mathrm{d}z + \int _{0}^\infty {\mathbb {E}}\left( \sup _{t\in [0,1]} \left\{ X(t)-|z|(1-t)\right\} \right) \mathrm{d}z. \end{aligned}$$

Now, by lower-bounding the supremum by taking only $t\in \{0,\tfrac{1}{2}\}$ and $t\in \{\tfrac{1}{2},1\}$ in the first and the second integrals, respectively, we obtain

$$\begin{aligned} {\mathscr {I}} \ge 2 \int _0^\infty {\mathbb {E}}\left( X(\tfrac{1}{2}) - \tfrac{1}{2}z\right) _+\mathrm{d}z \end{aligned}$$

which is positive because the integrand in strictly positive for $z>0$. $\square$
